# Results of a Community Randomized Study of a Faith-Based Education Program to Improve Clinical Trial Participation among African Americans

**DOI:** 10.3390/ijerph13010041

**Published:** 2015-12-22

**Authors:** Paula M. Frew, Jay T. Schamel, Kelli A. O’Connell, Laura A. Randall, Sahithi Boggavarapu

**Affiliations:** 1 Department of Medicine, Division of Infectious Diseases, School of Medicine, Emory University, Atlanta, GA 30322, USA; jayschamel@gmail.com (J.T.S.); laura.randall@emory.edu (L.A.R.); boggavarapu.sahithi@gmail.com (S.B.); 2 Department of Behavioral Sciences and Health Education, Rollins School of Public Health, Emory University, Atlanta, GA 30322, USA; 3 Hubert Department of Global Health, Rollins School of Public Health, Emory University, Atlanta, GA 30322, USA; 4 Department of Biostatistics, Rollins School of Public Health, Emory University, Atlanta, GA 30322, USA; kelli.oconnell@emory.edu; 5 Department of Epidemiology, Rollins School of Public Health, Emory University, Atlanta, GA 30322, USA

**Keywords:** health disparities, clinical trials, churches, study recruitment, African Americans

## Abstract

This is a report of a cluster randomized clinical trial evaluating the effectiveness of a church-based educational intervention aimed at improving African Americans’ (AA) participation in clinical trials. Two hundred and twenty-one AA subjects ages ≥50 years from six predominantly AA churches were randomized to intervention or control condition. The intervention included three educational sessions about clinical trials and health disparities; control participants completed questionnaires. Primary endpoints of the study were differences in individual subjects' intentions to obtain clinical trial information and intention to join a clinical trial, as determined by 10 point scale items at baseline, three and six months. A statistically significant increase in the intention to obtain clinical trial information at the three and six month time points was observed in the intervention group, but not the control group. Older participants (65–95 years) were less likely than younger participants (50–64 years) to increase their motivation to seek clinical trial information by the three and six month time points. No significant increases were observed in intention to join clinical trials. This randomized trial shows that AA church-based educational interventions are likely to increase the motivation of AA subjects to obtain clinical trial information and are therefore potentially effective at ameliorating the underrepresentation of AA subjects in clinical trials.

## 1. Introduction

Despite decades of significant medical advances that have resulted in approval of novel prevention strategies, therapeutics, and medical devices, clinical trials continue to be challenged by underrepresentation of racial and ethnic minority participants [1,2]. Recent data demonstrate that overall participation rates among racial/ethnic minorities continue to lag behind those of other groups for various types of clinical trials [1,3,4]. Moreover, enrollment rates are lower among the elderly (typically defined as those ≥70 years) when compared to younger participants [5,6,7], and lower among women than men in cardiology trials [1,4]. Older minority populations (age ≥65 years) carry a heavy burden of chronic and infectious disease morbidity and mortality, and are likely to be prime beneficiaries of medical advances if clinical trial results yield generalizable findings for this group [6,8]. Thus, underrepresentation of this group in trials presents a significant issue given the dramatic increases in the aging population that is also a growing consumer segment for pharmaceuticals and medical products [9,10].

With an increasing aging minority population, it is critically important that randomized clinical trials include adequate representation of this segment of the population [6,11]. Thus, underrepresentation has become an issue of social justice due to the potential for realization of significant health inequities in the years ahead [12,13]. Although older persons (ages ≥65 years) comprise 14% of the developed world’s population, this group also consumes one-third of pharmaceutical products, most of which were tested with non-representative populations [14]. Enrollment of persons ≥65 years, including racial and ethnic minorities, in clinical trials is therefore of global interest to address health disparities and the achievement of Healthy People 2020 objectives [15,16]. For this reason, the Food and Drug Administration (FDA), the U.S. Centers for Disease Control and Prevention (CDC), and other groups are now strongly advocating for reduction in age-based exclusions when feasible in clinical trials [16,17].

Significant challenges have been previously described in the recruitment of minorities including older adults (50–69 years) and elderly populations (≥70 years) [10,18,19]. These include logistical challenges such as getting to clinical locations, a lack of social support and social norms promoting participation, experiences of perceived stigma in medical environments, researcher distrust, health challenges, and adequate compensation [20,21,22,23,24]. Among those who have examined factors associated with clinical trial participation, perceived health status and personal/social benefit associated with enrollment factored as strong behavioral predictors [25]. In addition, simply knowing about studies recruiting the population has facilitated involvement of older adults, especially if communication was with a family member or health navigator [26]. Thus, awareness of trials open to enrollment and the personal relevance of the health topics addressed by available clinical studies, combined with perceived social support, have been demonstrated as important facilitators for engagement of this population [26].

Other studies have also highlighted the role of African American churches in recruitment of Southern African Americans, as this influence extends beyond religion [27,28]. For many older African Americans, the church is the centerpiece of religious, social, and political life. Additionally, the church provides an effective means to involve Southern African Americans in clinical research, especially women who belong to communities of faith, as they have been shown to be receptive to health messages delivered in this setting [29,30]. With the support and involvement of pastors, community members, and subject matter experts, we developed and tested an intervention entitled “Delivering a Dose of Hope” to address the problem of clinical trial underrepresentation among a specific segment of African Americans [29].

## 2. Experimental Section

### 2.1. Design

This study employed a cluster randomized study design to evaluate an educational intervention developed to increase clinical trial participation among African Americans aged 50 years and older [29]. Twenty churches in the Atlanta metropolitan area with ≥30% membership of African American congregants aged 50 and older were identified through ethnographic observation and informant interviews. The identified churches were enumerated based on denomination and estimated congregational membership to allow for matched pair selection. Three pairs of churches were then randomly selected to participate. One church in each pair was assigned to the control group while its match received the intervention.

Following church selection, study participants for each arm were recruited from their respective churches. Within churches, recruitment occurred through flyers, outreach, and health minister or pastor referral to the program staff for eligibility screening. To be considered for inclusion in the trial, an individual had to be ≥50 years, identify as Black/African American, and may not have previously participated in any clinical trials. Based on these criteria, we obtained written consent from 221 persons who were eligible to participate.

### 2.2. Participants

A total of 221 subjects between the ages of 50 and 95 were recruited from the six churches. Of these, 109 participants were in the control group and 112 participants were in the intervention group.

### 2.3. Intervention

Members of the intervention group participated in three information sessions about clinical trials and related health issues guided by church leaders, subject matter experts, and clinical researchers. The church leaders included health ministers with medical degrees and/or doctorates. We also invited Center Disease Control (CDC) public health practitioners with doctorates to speak. They developed PowerPoint presentations and handouts, and created interactive group exercises. Our clinicians and public health experts included faculty and staff from the Emory School of Medicine and Rollins School of Public Health and the Grady Healthcare System who held medical (MD) and public health (PhD and MPH) degrees. Special care was taken to recruit racially and ethnically diverse “program faculty” from within and outside the churches to develop and present the material in the designated three-hour time frame per session. Each presentation lasted up to 40 min with approximately 20 min of dedicated group discussion time for that topic with the speaker/subject matter expert program faculty. The sessions included discussions on lack of community participation in clinical trials (and related historical abuses), concerns about participant safety, clinical trial ethics, the influence of social networks, and health concerns relevant to the participants for which clinical trials were available for enrollment [29]. 

Control group participants completed questionnaires. They did not engage in any information sessions regarding enrollment in clinical trials, but were invited to attend community events such as health fairs and screenings, and educational presentations on health topics unrelated to clinical trials (e.g., mammography screening). Participants in both groups were notified of studies currently recruiting in the community through monthly telephone and email outreach.

### 2.4. Survey Measures

All study participants also completed questionnaires on attitudes, beliefs, and perceptions towards clinical research upon enrollment, and at three and six months after enrollment. The questionnaires consisted of items related to demographics, relationships with healthcare providers, attitudes about influenza and immunization, social ties and channels of communication, and attitudes, beliefs, and perceptions towards clinical research and participation in clinical trials. Study participants’ clinical trial enrollment was followed for an additional 18-month period after the six-month questionnaire.

As the primary endpoints, this interim analysis focused on the effect of the intervention on participants’ self-reported intentions to contact Emory University’s clinical study sites for information about clinical trials and intentions to join clinical trials. Final trial enrollment outcomes, and the effects of attitudes, beliefs, and social networks will be assessed in future studies. The primary hypothesis for this interim analysis was that the intervention would lead to increases in participants’ intentions to seek information about and join clinical trials. In future analyses, we will examine how these intentions translate into action.

### 2.5. Measures of Intention to Participate in Clinical Trials

We examined the intervention’s effects on two trial participation intention measures: (1) participants’ self-reported intention to seek information about clinical trials and (2) their intention to participate in clinical trials. Intention to participate in clinical trials was measured through two survey items: (1) intention to contact Emory University about participation in clinical trials and (2) intention to join a clinical trial, each measured at baseline, three months, and six months. Intention to seek information was measured by the question “On a scale from 1 (definitely not) to 10 (definitely so), rank your likelihood of *contacting* Emory University about being in a medical research study in the next 6 months,” where participants circled the appropriate number. Intention to join was measured with the question, “On a scale from 1 (definitely not) to 10 (definitely so), rank your likelihood of *joining* a medical research study within the next 6 months.” On the three- and six-month surveys, participants were asked whether they had sought information about clinical trials or joined a clinical trial since the previous survey. Those who reported seeking information about clinical trials were not asked their intentions to seek information, and were instead assigned a likelihood of 10 for intention to seek information for the appropriate time point. Similarly, those who reported that they had enrolled in a clinical trial at three or six months were assigned a likelihood of 10 for intention to join a clinical trial.

### 2.6. Statistical Analysis

Statistical analyses were performed using SPSS version 22.0 for Windows (IBM SPSS Inc., Chicago, IL, USA). Descriptive statistics were calculated for all variables, including means and standard deviations for continuous variables and frequencies and percentages for categorical variables. Chi-square and Mann-Whitney U-tests of independence were used to assess significant differences in sociodemographic variables between the control and intervention groups. Multivariable linear mixed effects models were used to examine the influence of the intervention on self-reported intention to seek information about and intention to join clinical trials while adjusting for several covariates, including age, gender, income, and baseline intentions. A single model was fitted to each outcome at each of the three-month and six-month time points, and included a random effect to account for the correlation between individuals within the same churches due to the cluster sampling methodology. Potential interactions between assigned groups and each of the covariates were examined, and significant interactions were included in the final model. Participants with missing outcomes or covariates were accounted for using casewise deletion.

Within the control and intervention groups, additional mixed effects models were used to examine changes in mean intention to seek information about or join clinical trials. These models adjusted for baseline intentions, age, and interaction between baseline intentions and intervention. Estimated marginal mean change in intention was calculated for each group and evaluated for significance using t-tests. All statistical tests were two-tailed and evaluated at a significance level of 0.05. Confidence intervals were calculated at a 95% significance level.

## 3. Results 

### 3.1. Sample Characteristics

A total of 221 subjects participated in the study, 112 in the intervention group and 109 in the control group. The mean age was 64.0 (SD = 7.7) and 78.7% (*n* = 174) of the participants were female (Table 1). Most participants had at least completed high school (94.1%, *n* = 208), with 64.2% (*n* = 142) having completed some form of post-secondary education. The majority of participants (66.1%, *n* = 146) had a household income of less than $60,000. Slightly less than half of all participants were married (46.2%, *n* = 102) and another 26.7% (*n* = 59) were divorced/separated. There were no significant differences in gender, education, or income between the control and intervention groups (Table 2). However, there was a statistically significant difference in the age distribution (Mann-Whitney U-Test; *p* = 0.03), with the control group having a significantly higher proportion of 70–79 year olds than the intervention group (z-test, *p* < 0.05).

**Table 1:** Sample characteristics.

Characteristics	*n*	%
Total	221	100
**Gender**		
Male	47	21.3
Female	174	78.7
**Age (Mean = 64, Median = 64, Standard Deviation = 7.7)**		
50–59	62	28.1
60–69	108	48.9
70–79	41	18.6
80–89	5	2.3
90–99	2	0.9
Missing	*3*	1.4
**Education**		
Grade K–8	3	1.4
Grade 9–11	10	4.5
High School/GED	66	29.9
Technical/Vocations/Associates	66	29.9
Bachelor’s Degree	37	16.7
Master’s Degree	33	14.9
Doctorate	6	2.7
**Income**		
<$20,000	61	27.6
$20,001–$40,000	49	22.2
$40,0001–$60,000	36	16.3
$60,001–$80,000	20	9.0
$80,001–$100,000	19	8.6
>$100,000	13	5.9
Missing	*23*	*10.4*
**Relationship Status**		
Single/Never Married	24	10.9
Married/Domestic Partner	102	46.2
Divorced/Separated	59	26.7
Widowed	35	15.8
Other	1	0.5
**Assigned Group**		
Intervention	112	50.7
Control	109	49.3

**Table 2:** Participant characteristics by assigned group.

Characteristics	Control		Intervention		
	N	% ^a^	N	% ^a^	*p*–Value ^b^
**Total**	109	49.3	112	50.7	
**Gender**					0.41
Male	22	20.2	25	22.3	
Female	87	79.8	87	77.7	
**Age**					0.03
50–59	25	23.1	37	33.6	
60–69	53	49.1	55	50.0	
70–79	29	26.9	12	10.9	
80–89	1	0.9	4	3.6	
90–99	0	0	2	1.8	
Missing	1	–	2	–	
**Education**					0.45
Grade K–8	1	0.9	2	1.8	
Grade 9–11	3	2.8	7	6.3	
High School/GED	33	30.3	33	29.5	
Technical/Vocations/Associates	31	28.4	35	31.3	
Bachelor’s Degree	23	21.1	14	12.5	
Master’s Degree	14	12.8	19	17.0	
Doctorate	4	3.7	2	1.8	
**Income**					0.50
<$20,000	32	33.3	29	28.4	
$20,001–$40,000	27	28.1	22	21.6	
$40,0001–$60,000	16	16.7	20	19.6	
$60,001–$80,000	8	8.3	12	11.8	
$80,001–$100,000	6	6.3	13	12.7	
>$100,000	7	7.3	6	5.9	
Missing	13	–	10	–	
**Relationship Status**					0.25
Single/Never Married	10	9.2	14	12.5	
Married/Domestic Partner	47	43.1	55	49.1	
Divorced/Separated	36	33.0	23	20.5	
Widowed	16	14.7	19	17.0	
Other	0	0	1	0.9	

^a^ Percentage of non-missing responses within study group. ^b^ Mann-Whitney U-test for age; chi-square test for all other variables.

### 3.2. Intention to Participate in Clinical Trials

**Figure 1:**
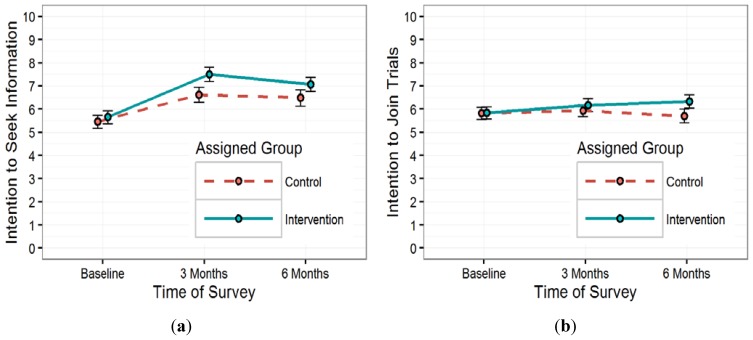
(**a**) Change in mean intention to seek information over time; (**b**) change in mean intention to join over time.

Changes over time in both mean intention to seek information about, and intention to join clinical trials for each group are shown in Figure 1. Baseline intentions to seek information about clinical trials were similar for the two groups (Figure 1a), with a mean baseline score of 5.5 out of 10 (SD = 2.9, missing = 2) for the control group, and 5.7 out of 10 (SD = 2.9, missing = 3) for the intervention group. At three months, the mean intention to seek information score for the control group was 6.6 out of 10 (SD = 3.3, missing = 4), while the mean score for the intervention group was 7.5 out of 10 (SD = 3.1, missing = 10). At six months, the control group had a mean score of 6.5 out of 10 (SD = 3.6, missing = 5) and the intervention group a mean score of 7.1 out of 10 (SD = 3.1, missing = 9).

**Table 3:** Intervention effect estimates and results from multivariable linear mixed models.

Predictor Variable	Intention to Seek Information				Intention to Join			
	3 Months *(miss = 42)*		6 Months *(miss = 43)*		3 Months *(miss = 41)*		6 Months *(miss = 44)*	
	β (95% CI)	*p*−value	β (95% CI)	*p*−value	β (95% CI)	*p*−value	β (95% CI)	*p*−value
Intervention (ref = control)	0.4 (−2.27, 3.08) ^a^	0.70	0.12 (−1.91, 2.16) ^d^	0.88	0.11 (−2.58, 2.35) ^b^	0.90	0.12 (−1.44, 1.69) ^d^	0.85
Baseline Intention	0.12 (−0.1, 0.34) ^c^	0.28	0.21 (−0.01, 0.43) ^c^	0.06	0.21 (0.02, 0.40) ^c^	0.03	0.12 (−0.11, 0.32) ^c^	0.33
Age	−0.09 (−0.15, −0.02)	0.01	−0.11 (−0.18, −0.05)	<0.01	−0.14 (−0.21,−0.07) ^d^	<0.01	−0.08 (−0.14, −0.02)	0.01
Gender (ref = male)	−0.83 (−1.96, 0.3)	0.15	−0.67 (−1.79, 0.45)	0.24	−0.76 (–1.68, 0.17)	0.12	−0.42 (–1.43, 0.59)	0.41
Income	0.09 (−0.21, 0.39)	0.57	−0.11 (−0.41, 0.2)	0.49	−0.04 (−0.29, 0.21)	0.76	−0.27 (−0.55, 0.001)	0.05
Intervention × Baseline ^e^	−0.14 (−0.44, 0.17)	0.38	−0.11 (−0.43, 0.2)	0.48	−0.12 (−0.40, 0.16)	0.39	−0.18 (−0.5, 0.13)	0.26
Intervention × Age ^f^	---	---	---	---	−0.11 (−0.22, −0.01)	0.04	---	---

^a^ The effect of the intervention when baseline is 7. ^b^ The effect of the intervention when age is 65 and baseline is 7. ^c^ The effect of baseline intention in the intervention group. ^d^ The effect of age in the intervention group. ^e^ Linear interaction of Intervention and Baseline Intention effects (that is, the amount by which the Intervention effect changes for each unit increase in Baseline Intention). ^f^ Linear interaction of Intervention and Age effects (that is, the amount by which the Intervention effect changes for each year increase in Age).

**Table 4:** Estimated marginal mean changes in clinical trial intentions.

	Mean Differences in Intention (95% CI) ^a^					
Assigned Group	Intention to Seek Information			Intention to Join		
	Baseline and 3 Months *(miss = 42)*	Baseline and 6 Months *(miss = 43)*	3 Months and 6 Months ^b^ *(miss = 48)*	Baseline and 3 Months *(miss = 41)*	Baseline and 6 Months *(miss = 44)*	3 Months and 6 Months (*miss = 46*)
Control	1.08 (−0.44,2.6)	1.02 (−0.43, 2.47)	0.07 (−0.76, 0.9)	0.09 (−1.15, 1.32)	−0.03 (−1.16, 1.1)	−0.18 (−1.54, 1.18)
Intervention	1.98 (0.47, 3.5) *	1.49 (0.05, 2.93) *	−0.29 (−1.11, 0.54)	0.36 (−0.86, 1.59)	0.52 (−0.61, 1.65)	0.22 (−1.14, 1.57)

**^a^** All models adjust for baseline intention, age, and intervention * baseline intention. ^b^ Used model without church random effect because estimated between-church variance was zero.

Intention to join clinical trials at baseline was similar for the two groups as well, with the control group having a mean baseline score of 5.8 out of 10 (SD = 2.7, missing = 4) and the intervention group a score of 5.8 out of 10 (SD = 2.7, missing = 3) (Figure 1b). At 3 months, the mean score for the control group was 5.9 out of 10 (SD = 2.7, missing = 4) and for the intervention group 6.2 out of 10 (SD = 2.8, missing = 9). The mean intention score at 6 months was 5.7 out of 10 (SD = 3.0, missing = 5) for the control group and 6.3 out of 10 (SD = 2.9, missing = 9) for the intervention group.

Results from the multivariable linear mixed models for three- and six-month intentions to seek information about and join clinical trials are shown in Table 3. Participation in the intervention group was not associated with greater improvement in intention to seek information about or join clinical trials at follow-up. Age was significantly related to three-month intention to seek information (*p* < 0.05), six-month intention to seek information (*p* < 0.01), three-month intention to join (*p* < 0.01), and six-month intention to join (*p* < 0.05), with older participants indicating less increase, or greater decrease, in intentions over baseline. There was a significant interaction between age and intervention in the three-month model for intention to join clinical trials: in the intervention group, younger age was significantly associated with more positive change in intention to join clinical trials at three months relative to baseline (*p* < 0.01), while in the control group, age was not significantly associated with a difference in the change in intention to join from baseline to three months (*p* = 0.51).

A summary of the estimated marginal mean change in the two trial participation intention measures within each group resulting from the multivariable linear mixed models for mean changes are presented in Table 4. In the intervention group, intention to seek information about clinical trials on average increased significantly from baseline to three months (adjusted mean difference = 1.98, *p* < 0.05) and from baseline to six months (adjusted mean difference = 1.49, *p* < 0.05), after adjustment for baseline intentions, age, and interaction between baseline intentions and intervention. Control group participants did not see a significant increase or decrease in intention to seek information about clinical trials, on average, between any pair of time points. There were no significant changes in the average intention to join between any pair of time points for either group.

## 4. Discussion

This study found that the comprehensive intervention “package” characteristics (*i.e.*, church-based, pastor-supported, based in social networks) likely resulted in the initiation of behavioral change consistent with established theoretical models and persuasion frameworks [31,32,33]. Health communication models indicate that persuasion occurs over a continuum and is bolstered by repeated exposure to messages or content [34]. According to previous behavioral communication studies, persuasion likely occurs at later time points after which the credibility of the source is established through information verification processes also known as “assimilation” [35,36].

The results from this study demonstrate that the first step in motivating people to consider participation—intention to seek information about clinical trials—increased significantly by three months in the intervention group, much faster than we anticipated. Thus, with our participants indicating that they were more likely to get information over this initial introductory program period, the findings offered evidence that information seeking would precede behavioral initiation accounting for the presence of “attitudinal ambivalence” [37]. This term refers to a condition we expected would be present due to knowledge of medical abuses via oral history or, for the intervention arm participants, stemming from our discussion of past clinical trials and ethics abuses in medical and public health research. Our participants were likely to feel conflicted about the information we would present, as their beliefs and knowledge would contribute to attitude formation and subsequent participatory decisions to join or forego joining clinical trials. In other words, we expected that we needed three sessions with our participants to establish our “source credibility” and, consequently, to generate any motivation to attend to messages we presented about clinical trials [36]. In reality, we needed only two sessions for participants to formulate their decision to seek additional information and reduce attitudinal ambivalence.

The study findings also indicate that intention to join clinical trials also seemed to increase by the three-month time point, yet the increase was not statistically significant. Similarly, the effects resulting from participation in the program diminished after three months. Both intention to seek information about and intention to join clinical trials did not increase significantly between three- and six-month time points. Indeed, previous studies have demonstrated that persuasion is most effective when study participants are exposed to strong messages, have time to think about them, and have the ability to process the message in the face of counter-persuasion (e.g., negative enduring beliefs and/or social norms about clinical trials) [34]. Our findings suggest that most processing likely occurred in the first three months of program involvement and tapered off after that time point.

Although our participants indicated their intention to seek more information about clinical trials at the three-month time point, there is insufficient evidence to attribute this observed, significant increase to the intervention itself. It is possible that a portion of this increase is attributable to positive interaction with the researchers, the perceived social influence of the church network involved with the study, and the availability of information about current clinical trials that was provided to both the control and intervention groups. As this program was developed in collaboration with our Community Advisory Board, pastors from participating churches and their health ministers, any effect may also be attributable in part to the Community-Based Participatory Research (CBPR) processes that occurred [38]. Indeed, there is a growing body of literature on specific persuasive effects that motivate older minorities to participate in research studies [39]. Specifically, hearing the recommendations of family members, physicians, and others, combined with perceived social support for participation, serve as powerful motivational queues [39]. Because control intentions also appeared to increase slightly (though not statistically significantly), we believe all of the above likely contributed to increased intentions; however, more experimental arms and additional research are necessary to assess these effects.

The findings also correspond with those of previous studies that point to significant challenges associated with the recruitment of elderly populations [10,19,26]. Advanced age (≥65 years) tempered the increase in intentions over the course of the study; elderly participants in both groups displayed smaller increases in intention (or greater decreases in intention) than their younger (50–64 years) counterparts on both intention outcomes. This suggests that our participants were well-aware of their personal limitations associated with participation, including reliance on others for transportation to/from study visits, their physical immobility, and their health restrictions likely resulting in their exclusion from studies. Thus, their intentions reflect an array of sociostructural and personal factors that must be considered in future interventions seeking to enroll this group [40]. We argue that a multi-pronged intervention which accounts for distance to/from clinical trial sites, transportation options, and whose study promotion messages originate from the church would characterize a future intervention model. In addition, it will be worth evaluating this time-intensive approach to other strategies, such as direct patient outreach and mass advertising to determine its cost-benefit ratio.

We acknowledge the limitations in this study. The self-reported intentions to seek information about or join clinical trials that are examined as the analytic outcomes are intermediate to the behavioral outcomes of information seeking and joining clinical trials. However, these intermediate outcomes provide important information about both the pathway to action for clinical trial participation and the internal processes that may lead to action. We also recognize that this pilot intervention is limited in its broader application based on these pilot results. Our interventions in urban Atlanta churches may yield different results than those performed in other cities or in rural areas, especially as the easy availability clinical trials relevant to the participants is necessary for intervention success. On-site enrollment and trial implementation could help improve participation where trial access is difficult [41]. We also know that the involvement of pastors and church leaders is critical to recruitment and to congregant engagement in the intervention, yet their influence may also introduce ethical concerns related to coercion. Our participating churches had a variety of educational backgrounds, and on average our participants had a relatively high degree of educational attainment, making results difficult to generalize to churches serving congregants with less formal education.

## 5. Conclusions

After three months, participants from the intervention arm showed increased intentions to seek information about clinical trials. These results suggest that the combination effect derived from positive engagement with researchers, health ministers, pastors, and other subject matter experts in a familiar faith-based setting, plus obtaining information about relevant clinical trials quickly, engages older African Americans on research decision-making. Because these increased intentions were not mirrored by participants of advanced age (≥65 years), it is likely that additional efforts must be made to address the special barriers to research participation faced by the elderly. Nonetheless, this study highlights the promise of church and faith networks as avenues through which to influence older African Americans’ knowledge and attitudes towards clinical research participation.
